# On the Good Effects of Carbonate of Magnesia

**Published:** 1810-06

**Authors:** 


### *To the Editors of the Medical and Phyfical Journal.*

GENTLEMEN,

THE good effects of carbonic acid when taken into the stomach is well known to most of the members of the medical profession. This, I believe, was first observed by Dr. Macbride, of Dublin, and soon afterwards by Dr. Percival of Manchester, who warmly recommended it as an antiseptic.

Its effects were still more promulgated by that eminent chemical philosoper Dr. Black, whose discovery it was, and who found it very useful in calculous complaints. The manner in which this is commonly exhibited internally, is by uniting the juice of a lemon with subcarbonate of potash, in sufficient quantity to saturate it, and this is to be taken in a state of effervescence. Others give it in a more diluted form, as an agreeable beverage in febrile diseases, but in this state it ceases to emit the carbonic acid.

As the good effects which proceed from this medicine are allowed to arise from the action of the carbonic acid on the nerves * of the stomach, and as lemons are now expensive, and even prohibited in some hospitals, I was led to search for a substitute for a remedy so valuable in almost all cases of irritation of the stomach, as well as in putrid diseases. Having reflected on the manner in which I could most conveniently introduce the gas into this viscus, well knowing its deleterious effects when inhaled, although Dr. Percival thought it a valuable remedy in phthisis, (which, no doubt, proceeded from its salutary operation on the stomach) I found the carbonate of magnesia, which contains more of this gas than the other alkaline

* Vide Black's Letters.

kaline earths, lime excepted,† to answer very well when united with distilled vinegar. This acid will be found sufficiently powerful for disengaging the carbonic acid gas, and is at the same time so innocent as to be taken with perfect safety. A scruple of the carbonate of magnesia saturates about an ounce of this acid, causing a violent emission of fixed air, which continues for some time. This is always to be used in a state of effervescence, as when diluted, it neither emits gas nor unites with the water.

This I have not only used with great success in all cases of irritation of the stomach, but more especially in continued fevers, where debility was very great, and where, from the disordered state of the stomach and alimentary canal, no other medicine could be retained. On being rec ived into this organ, it not only remains, but the patient experiences an agreeable sensation from it, often superseding the necessity of the accustomed drink. I have had numerous oppoitunities in the military hospital of witnessing its good effects; and being myself subject to a dyspeptic complaint for some time, which was very much increased by inanition, and having tried purging, vomiting, and a number of bitters and astringents, I was induced to ascertain its effects. This remedy I took when the pain was very great, and it had no sooner entered the stomach than it caused several acid eructations, after which I became quite easy. This I have often repeated with similar results.

Of the success of this composition I have had ample experience in many other dyspeptic complaints; but as enough has been said above, I only request that some of your practical Readers may be induced to give it a trial. They will, at least, find in it an excellent substitute for the common effervescing draught, and it may always be had recourse to, when lemons cannot be obtained.

I am, &c.

W. H.

*Ipswich New Barracks,*

*May* 7, 1810.

---

† Carbonate of magnesia, as obtained in commerce, does not contain a maximum of acid, it is only by a subsequent process it is ever fully saturated with the proportion of acid mentioned by the author. Ed.

